# UHRMS Formula Assignment: Diophantine-Based Recalibration Yields Lorentzian Mass Error Distribution as the Limiting Factor

**DOI:** 10.1021/jasms.5c00226

**Published:** 2025-12-26

**Authors:** Neda Safaridehkohneh, Albrecht Ott

**Affiliations:** Department of Physics, Center for Biophysics, 9379 Saarland University, Saarbrücken 66123, Germany

## Abstract

Ultrahigh-resolution mass spectrometry (UHRMS) is a well-established analytical method for characterizing complex molecular mixtures. It is usually performed with Fourier transform techniques, based either on ion cyclotron resonance (FTICR-MS) or mass-dependent oscillations in an ion trap (FT-Orbitrap-MS). In spite of the high technical level of these instruments, often spectral interpretation remains difficult, in particular in a nontargeted approach of complex samples. Here, we introduce a Diophantine method for molecular formula assignment. Taking the ubiquitous Gaussian distribution as an example, we first show how knowledge about random mass error can be used to assign molecular formulas in a statistically consistent way. By considering all possible attributions within a large mass error range, we show how the systematic error stemming from suboptimal calibration can be distinguished from the random mass error in peak position. Correcting for systematic mass error leaves us with a quantifiable, Lorentzian random mass error as expected for Fourier transform-based instruments with long transients. This indicates that our method is self-consistent, assigning molecular formulas close to the theoretical limit of achievable accuracy.

## Graphical abstract

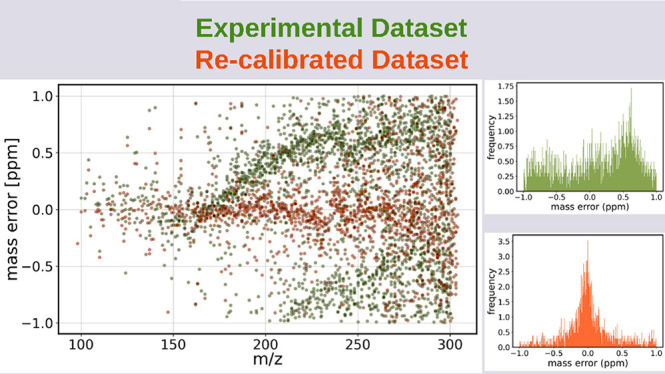

## 1 Introduction

Ultrahigh-resolution mass spectrometry (UHRMS), typically exceeding a resolving power of 100,000, is performed on Fourier transform ion cyclotron resonance (FTICR-MS) and Fourier transform Orbitrap (FT-Orbitrap) instruments. It is a powerful analytical tool, widely used to characterize complex chemical mixtures from various sources, including the environment, biological matter, synthetic compounds, pharmaceutical products, as well as the petroleum industry^1–5^ . UHRMS promises to identify, in a single measurement, a huge number of molecular compounds (up to a few ten thousands) within a complex sample. This type of information can be crucial, for instance, in nontargeted assessments of toxicity of intricate molecular mixtures. It can be of great help in prebiotic scenarios^6^. This is among many other settings that concern complex mixtures of molecules in industrial applications or scientific research.

A significant large-scale effort to evaluate the reproducibility of measurements and assignments by different groups with different instruments highlighted the critical need for reliable assignment methods^7–11^ . Interpreting complex spectra in a nontargeted approach remains challenging. Several advanced molecular formula assignment methods have been developed. These include licensed based methods (e.g., Composer from Sierra Analytics, PetroOrg^12^) as well as open-source database related methods (e.g., UltraMassExplorer (UME),^13^ FuJHA,^14^ Formularity^15^). However, completeness and correctness of the database are crucially important. Up to 30% of spectra in the NIST EI mass spectral database were claimed to be incorrectly assigned^16^. There are also open-source calculation methods (e.g., CHO-FIT^17–19^ ) that do not require a database. A strong limitation in the assignment process stems from the combinatorial explosion accompanying higher mass molecules made of several atomic species, making purely calculative approaches challenging. Graph theoretical - and Diophantine approaches have yielded elegant methods for improvement^20–22^ . The CHOFIT method^17,18^ used low mass moieties limited to CHO, leading to excellent results on small molecules.

Despite the high precision of UHRMS techniques and devices, incorrect calibration often remains a significant source of error that complicates accurate assignment. It is advised to use internal calibration standards to control deviations that may occur due to space charges in the cyclotron (or the trap)^23,24^ . Space-charge effects in FT-ICR cause frequency shifts that depend not only on total ion abundance but also on ion cloud interactions, which can be linked to local peak densities and amplitudes^25–27^ so that corrections can be performed^26^. In Orbitrap instruments, space-charge effects can be pronounced due to ion-cloud interactions and field nonidealities^28,29^ . Local ion–ion interactions can lead to peak coalescence so that closely spaced ion peaks merge into a single detected peak. This effect is particularly pronounced if the relative abundance ratios are high^29^, causing the weaker ion cloud (the weaker peak) to lose coherence and collapse into the stronger one. This has been experimentally observed on Orbitraps^29^ and FT-ICR instruments^30,31^ . As a result, additional measurement strategies can be useful to improve accuracy beyond ppm error. Segment-based approaches like OCULAR hold great promise in complex mixtures, where they improve the number of detected substances as well as the accuracy of the determined *m*/*z* as compared to simple, one shot injection^32^.

Error and noise are an inherent parts of every data acquisition process. The finite residence time of ions in the trap represents a physical limitation causing an exponentially decaying signal in the time domain resulting, due to the Fourier transformation, in a Lorentzian peak shape in the frequency domain. If the signal is cut off before decay, a sinc-shaped peak will appear. The peak shape directly corresponds to the theoretical error distribution caused by the (unavoidable) limitation in data^23^. Moreover, weakly concentrated ions will exhibit more random peak shapes due to the limited statistics that come with a vanishing number of ions^33^. Other sources of mass inaccuracy, for example from detection electronics, may increase error, although in well-designed instruments, they should not contribute by much^34^. Mathur^35^ showed that the quality of the preamplifier sets the mass error of the baseline, which limits the instrument in the detection of weakly concentrated ions. For Q-TOF mass spectrometry, different methods have been developed to model and denoise acquired data^36–39^ ;their systematic application in FT-based mass spectrometry remains limited. To our knowledge, for FT mass spectrometry, the impact of noise and error on the accuracy of assignments has not yet been fully worked out.

In this study, we use linear Diophantine equations for assigning chemical formulas in a combinatorial way to data from UHRMS. However, instead of iteratively assigning peaks and checking the plausibility of the quality of the assignments at the end by looking at the statistics of mass deviations, here we take the opposite route. We start by considering all possible assignments (correct or not) that fall within a suitable range of mass deviation. As their mass deviation is plotted as a function of *m*/*z*, due to the nature of the solutions to the Diophantine equations, a characteristic, symmetric pattern must appear. We first investigate up to which point an intrinsic, random mass error from the instrument limits the assignments in a perfectly calibrated setting. We then show that deviations due to imperfect calibration can be spotted and corrected for, since a correct calibration necessarily aligns with the symmetric Diophantine pattern. We apply our method to data from a prebiotic broth generated in a Miller-Urey type experiment and analyzed on a Bruker Solarix FTICR-MS (without apodization) using electrospray ionization (ESI). We recalibrate the measurement to a better degree than what is achieved by internal calibration to obtain a basically Lorentzian distribution of the mass error as the limiting factor for assignment. It is this error that needs to be taken as the intrinsic error for a statistically meaningful assignment.

## 2 Background: Diophantine Equations

Diophantine equations, a class of equations requiring integer solutions, have already been discussed in the context of mass spectrometry^40^ . They provide a natural framework for molecular formula assignment, since each measured nominal mass (the integer of its exact mass) must equal the sum of integer multiples of the nominal masses of the constituent atomic species. Usually, the uncertainty of the instrument is such that the measurement of the nominal mass is not affected by error. Accordingly, the molecular formula of the detected nominal mass *M* must be among the solutions of the Diophantine equation:

$$\sum_{i}n_{i}*M_{i}=M$$ 1

where *i* indicates the different atomic species under consideration; *n*_*i*_ is the integer count of species i; and *M*_*i*_ is its nominal mass. This formulation follows from the discrete nature of atoms, which defines the problem as Diophantine. Unlike classical enumeration-based approaches, this formulation generates candidate formulas systematically through combinations of so-called “special” and “homogeneous solutions”.

A special solution of the Diophantine equation can easily be obtained as follows.

**(i) carbon count:** calculating the count of carbons through integer division of *M* by the mass of carbon:

$$n_{C}=M\mathrm{div}\;12$$ 2

**(ii) hydrogen count:** the number of hydrogen atoms can be obtained as the remainder of this division:

$$n_{H}=M-12\times \left(M\mathrm{div}\;12\right)$$ 3

This pair, (*n*_*C*_, *n*_*H*_), serves only as a mathematical starting point. In some cases, for example *M* = 72, the special solution yields only (six) carbons and no hydrogens. Our algorithm adjusts such cases (e.g., by subtracting one carbon and adding 12 hydrogens) before proceeding.

Even for more than two atomic species, all solutions that yield the nominal mass *M* can be obtained from one special solution (any special solution will do) by adding all solutions of the homogeneous Diophantine equation:

$$\underset{i}{\sum }n_{i}\cdot M_{i}=0$$ 4

where *n*_*i*_ are integers that can be positive or negative, and *M*_*i*_ is the nominal mass of the atomic species *i*. Note that the exact masses corresponding to the solutions of eq 4 are nonzero, so that the elements of the set of Diophantine solutions (the set of possible assignments for the nominal mass *M*) differ in their exact masses. To give an example, possible solutions to the homogeneous equation include *C*_4_ *O*_–3_ and *C*_–4_ *H*_2_ *O*_2_ *N* with exact masses of 1.5255 × 10^–2^ Da and 8.554 × 10^–6^ Da, respectively.

Solutions of the homogeneous equation form a vector space with integer coefficients, spanned by *A* – 1 base vectors where *A* is the number of atomic species i that come into play (see SI, “Diophantine Vector Space” for details). Each base vector *v*_*i*_ has a nominal mass of zero, but an exact mass *m*_*v*_*i*__ ≠ 0.

For formula assignment, the exact mass of the special solution, $M_{\left(C_{n_{C}}H_{n_{H}}\right)}$ , is compared to the measured mass *M*_*M*_:

$$\Delta M=M_{M}-M_{\left(C_{n_{C}}H_{n_{H}}\right)}$$ 5

The goal is to find integer coefficients λ_*v*_*i*__ to match |Δ*M*| within the error δ that must be chosen as a function of the accuracy of the measurement:

$$\Delta M-\delta <\underset{i}{\sum }\lambda_{v_{i}}m_{v_{i}}<\Delta M+\delta$$ 6

All possibly assigned molecular formulas are given by the special solution plus homogeneous solutions from linear combinations of base vectors λ_*v*_ *v* that satisfy the condition in eq 6.

### 2.1 Example

Consider a detected, protonated ion with *m*/*z* = 90.05495547 Da. The corresponding neutral mass is 89.047679 Da, with a nominal mass of 89 Da. A special solution is C_7_H_5_, with an exact mass of 89.039125 Da. The mass deviation from the neutral mass is Δ*M* = 89.047679 – 89.039125 = 0.008554 Da. Within one ppm tolerance, all solutions to eq 4 in the range (8.9 × 10^–5^ ± 0.008554 Da) are considered. The best elemental match is C_–4_H_2_O_2_N (8.554 × 10^–3^ Da). Adding this to the special solution reveals C_3_H_7_O_2_N, corresponding to Alanine.

**2.2 Significance of the parameter δ.** Note that δ in eq 6 sets a finite volume in vector space, in other words, a finite number of possible solutions/assignments (at least if we impose limits on the number of atomic species). We understand that this volume increases as a power law with the error of the measurement with an exponent that depends on the number of elements under consideration. However, the number of possible assignments increases exponentially (or geometrically) with the number of atomic species. If the instrument maintains a certain error in terms of ppms across the considered mass range, the absolute value of δ increases proportionally to the mass under consideration, that is, the number of possible assignments grows as a power law. Unfortunately, ICR and Orbitrap instruments often show an increasing relative error with mass^41,42^ . As a result, the number of possible assignments grows faster than polynomially with *m*/*z* and faster than exponentially with the number of atomic species. However, some instruments maintain a roughly constant relative error across the considered *m*/*z* range^43^. We understand that the accuracy represented by the parameter δ is crucial; however, a priori, the accuracy of the measurement is unknown since many parameters may contribute to the deviation of the measurement from the ideal case.

## 3 Computational Details

### 3.1 Optimized Implementation of the Diophantine Algorithm

To speed up computation, we do not simply apply the condition in eq 6. Instead, we first compute a library of solutions to the homogeneous Diophantine equation (eq 4). The library is limited to positive exact masses, sorted in ascending order. The library consists of low-mass compounds containing carbon, hydrogen, oxygen, nitrogen, sulfur, and phosphorus, limited as follows: |C|≤ 40, |H|≤ 140, |O|≤ 20, |N|≤ 12, |P|≤ 8, |S|≤ 8. The limits on each element were chosen based on known sample composition, and a trial-and-error process: we adjusted the limits to ensure that every plausible compound could be generated in the library while keeping the computational load manageable. These constraints are in line with the “Seven Golden Rules”^44^. We refer to the elements of the library as low-mass moieties (LMMs) that occupy the negative van Krevelen space.^17^

With these limitations on LMMs, we can find an assignment for masses up to 1500 Da within the defined accuracy. Here, we only consider masses up to 500 Da for synthetic data and up to 300 Da for experimental data. Therefore, the limitations imposed on LMMs should not matter for the cases considered. To generate all exact masses within the tolerance δ of the measured mass following eq 6, we extract the corresponding subset of LMMs from this library.

### 3.2 Isotope Pair Detection and Filtering

Accurate recognition of isotopes is essential for molecular assignments. Here we present a method limited to carbon that is valid for small molecules. Other isotopes have a low probability of appearing in our case so that we can safely neglect them. For cases with a larger probability of more complex isotopologues, more sophisticated algorithms such as IsoSpec need to be applied in spite of the increased computational expense^45,46^ . Electrospray is used for ionization in our instrument. It is known to produce almost only single charged molecules for small masses^47^ so that we can safely neglect multiply charged molecules.

The isotope filtering process detects paired isotopes in the input mass list. It involves three steps:

**(i) Setting the search interval** δ*M*: for a given mass *M*_1_ from the input list, masses within the range of *M*_1_ + 1.003355 ± δ*M* are selected, as shown in Figure 1. δ*M* represents the half-confidence limit based on the expected accuracy of the measurement. By default, we use δ*M* = 0.0001678 Da. The value of 1.003355 corresponds to the mass difference between ^12^ *C* and ^13^C.

**Figure 1:**
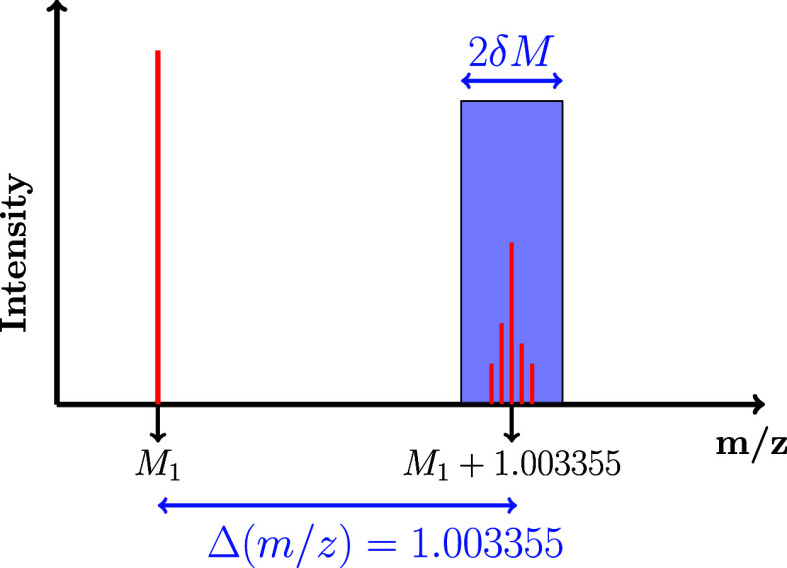
Isotope filtering process. The *x*-axis represents the mass-to-charge ratio. The *y*-axis indicates the peak height of detected masses in the mass spectrum. 1.003355 corresponds to the mass difference between ^12^C and ^13^C. For a given mass, *M*_1_, a paired isotopic compound must be expected at *M*_1_ + 1.003355. With δ*M*, the half-confidence limit of the measurement, all masses within *M*_1_ + 1.003355 ± δ*M* (blue rectangle) are considered to be possible isotopes of *M*_1_.

**(ii) Kendrick Mass Defect (KMD) Analysis:** Here, KMD refers to a generalized transform used for isotope detection rather than traditional CH_2_ homologous series analysis. The base unit is set to the ^13^C – ^12^C mass difference (Δ*m* = 1.003355 Da), so that isotopologue series align. This isotopic KMD is applied to flag possible poly isotopic peaks prior to further checks and formula assignment. This saves computational time. As an example, the KMD of a mass *M*_1_ = 235.18064 Da is calculated as follows:

$$\begin{array}{rll} (\mathrm{KMD}{)}_{M_{1}} & =\frac{M_{1}}{1.003355}-\left\lfloor \frac{M_{1}}{1.003355}\right\rfloor & \\ & =\frac{235.18064}{1.003355}-\left\lfloor \frac{235.18064}{1.003355}\right\rfloor & \\ & =234.394247-234=0.394247 & \end{array}$$

where ⌊.⌋ represents the rounding down function. Two masses are considered a match if their KMD values are identical to the fourth digit, corresponding to a tolerance of approximately 0.067 ppm (see SI, “Isotope Filtering Tolerance Calculation”, for details).

**(iii) Relative Intensity Analysis:** This is a postfiltering analysis that is performed after assignment. The peak heights of the isotope candidates are checked against the expected abundance ratios. Here, we allow for a deviation of 10*%* in the logarithm of relative peak heights. This deviation must be set depending on the response function of the instrument.

A polyisotopic pair is confirmed if it meets all the three conditions above. See SI under “Isotope Filtering and Relative Intensity Analysis” for details on checks of the relative heights of isotopic peaks, including an exemplary calculation.

### 3.3 Selecting Chemically Plausible Formulas Using the Seven Golden Rules

Compared to a purely combinatorial approach, many chemically nonmeaningful molecular formulas can be excluded by applying six of the seven Golden Rules^44^. The seventh rule is specific to small molecules in clinical and metabolomics studies using GC/MS, beyond the scope of this work, so, we dropped it.

The rules include: (i) restriction of element counts, (ii) compliance with LEWIS and SENIOR rules, (iii) verification of polyisotopic patterns, (iv) assessment of hydrogen-to-carbon ratios, (v) evaluation of nitrogen, oxygen, phosphorus, and sulfur to hydrogen ratios, and (vi) checks for high-probability, multiple element-to-carbon ratios.

Moreover, elemental ratios are limited as follows: H ≥ 2, $\frac{H}{C}<3.1$ , $0\leq \frac{O}{C}<3$ , $0\leq \frac{N}{C}<1.3$ , $0\leq \frac{P}{C}<0.3$ , and $0\leq \frac{S}{C}<0.8$ . Table 1 summarizes the numeric constraints on the presence of multiple elements within a molecule.

**Table 1:** Constraints on Multiple-Element Numbers for Compounds up to 2000 Da as Adapted from Kind et al^44^.

elements	constraints
N, O, P, S > 1	N < 10, O < 20, P < 4, S < 3
N, O, P > 3	N < 11, O < 22, P < 6
O, P, S > 1	O < 14, P < 3, S < 3
N, P, S > 1	N < 4, P < 3, S < 3
N, O, S > 6	N < 19, O < 14, S < 8

### 3.4 Algorithm and Computational Details

Figure 2 illustrates the algorithm. It starts by setting key parameters, such as the mass interval for isotope filtering, peak height deviation tolerance for isotopic pair verification, and mass deviation tolerance for assignments. These parameters can be modified by the user as a function of requirements. It continues by detecting polyisotopic pairs. The input data in the form of a list is divided in two: paired and unpaired masses. These two sublists are then processed separately using the Diophantine algorithm for molecular formula determination. Candidate formulas are checked against six of the seven Golden Rules. Formulas meeting these criteria are stored in the output list for further analysis. This implies that, for a single input mass, most of the time multiple possible molecular formulas are suggested.

**Figure 2:**
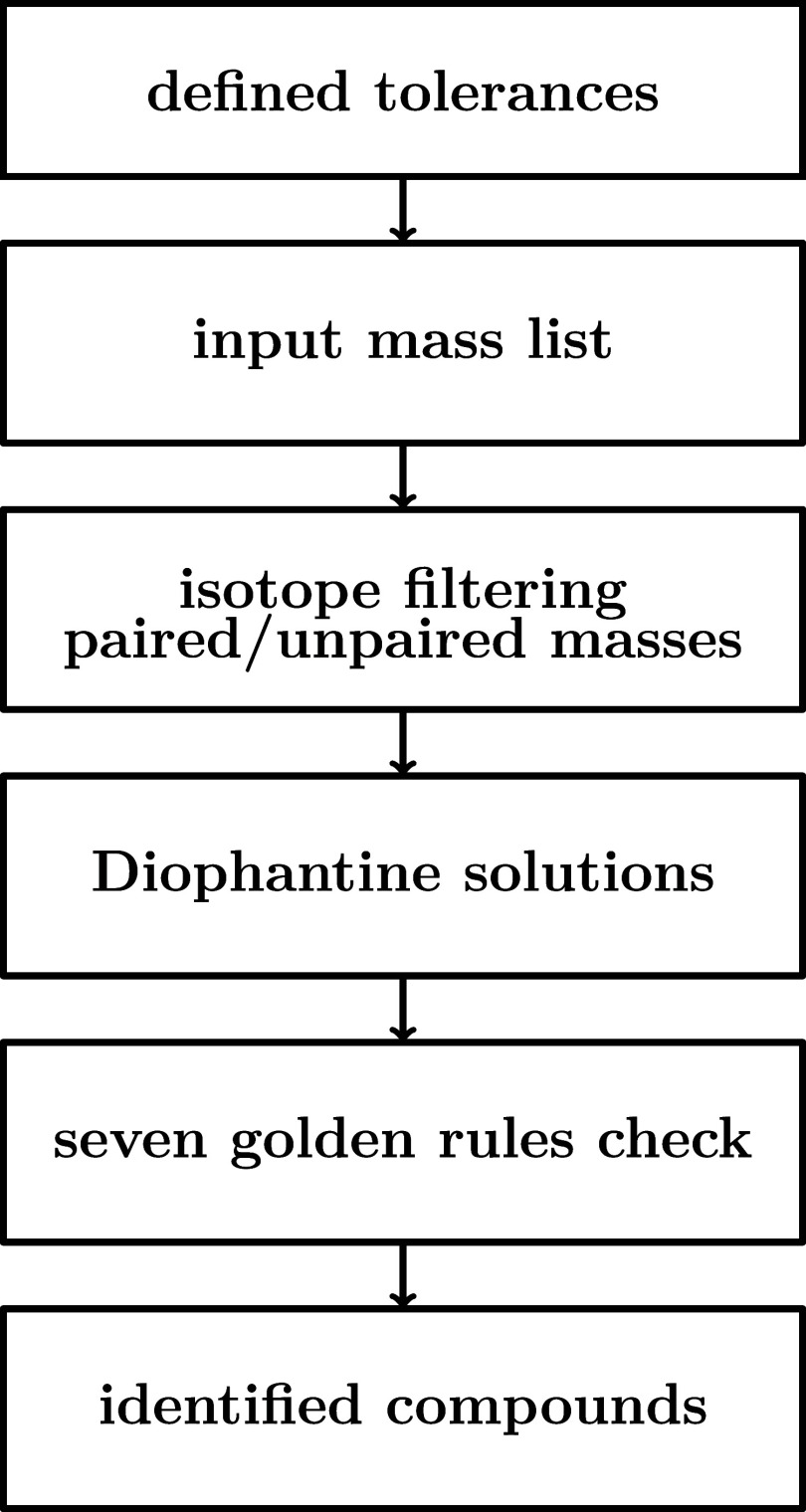
Flowchart of the proposed algorithm: after parameter initialization, the *m*/*z* list is isotope-filtered into paired and unpaired subsets. Both undergo Diophantine assignment, generating candidate formulas filtered by six of the seven Golden Rules. Chemically plausible formulas are stored in the output.

### 3.5 Outline of the Calibration and Assignment Process

Classical molecular formula assignment algorithms often rely on some form of recalibration, either by identifying internal calibrants or using an algorithm, for example the CHOFIT method, which tries to find the molecules made only from CHO, in the hope of attributing this limited amount of molecules correctly^17^. This is followed by brute-force enumeration of all possible combinations of elements up to certain limits, followed by heuristic filters such as the “Seven Golden Rules”^44^. This produces a figure of merit, where the mass deviation of the attributions is compared to a Gaussian error distribution with an error usually below one ppm. In contrast, our approach is explicitly rooted in the mathematical structure of Diophantine equations. For calibration, we know that we eventually need to generate a symmetric pattern if we plot the mass deviation errors of *all* possible assignments (including candidate assignments beyond the correct ones) as a function of *m*/*z*. The symmetry is because of the homogeneous solutions that can be added or subtracted from the correct assignment represented by the axis of symmetry. The pattern emerges from the discreteness of the Diophantine solutions. We then extract an error from the calibrated data. This error should be fed into the assignment process as detailed in the first part of this paper to perform a consistent assignment. With that, the fraction of false attributions is known. Our method is simple and fast compared to any other methods that we have found. It yields results close to what is technically achievable, given the data as delivered by the instrument.

## 4 Methods

### 4.1 Hardware

We used a personal computer with a Linux (Ubuntu) operating system, featuring a 12th generation Intel Core i7 processor capable of operating in 32 GB and 64 GB modes, running at 2.0 GHz. The algorithm was programmed in house using Python (3.10.12). Each run of assignments for a mass list between 150 and 500 Da typically required two to 3 h of time, not including the setup of the library. A data set with imposed random Gaussian mass error required more computational time due to increased complexity.

### 4.2 Data Sets

#### 4.2.1 Synthetical Data Set from Green and Perdue

The mass list from Green and Perdue^17^ comprises combinations of isotopes of carbon, hydrogen, oxygen, nitrogen, sulfur, and phosphorus, spanning masses from 150 to 1500 Da. For practical purposes, we restricted the mass range to 150–500 Da.

#### 4.2.2 Adding Mass Error and Decalibration

We added Gaussian mass error to the synthetical data set. The Gaussian mass error distribution is characterized by zero mean and a defined standard deviation, quantifying the mass error level. For a given exact mass, *M*, Gaussian mass error produced a statistical error Δ*M* resulting in a new mass value *M′* = *M* + Δ*M*. Δ*M* can be positive or negative. The standard deviation was set to an average value expressed as parts per million (ppm). Note that since the standard deviation was fixed in absolute terms, the error expressed in ppm was higher for small and smaller for large *m*/*z*. Setting the error in absolute or in relative terms is not expected to affect our conclusions. In practice, the error and its dependence on *m*/*z* is a function of the instrument and needs to be determined from the data (cf. Figure 12 for an example).

We decalibrated the data set with added Gaussian mass error using a quadratic polynomial, inspired by the classical quadratic calibration equation of Ledford and Gross^48^.

#### 4.2.3 Experimental Data Set

We performed mass spectrometry on a prebiotic broth, prepared as described in^6,49^ . The sample volume, a few ml, was filtered with 2 μm pore size before measurement. We performed direct injection using ESI on a Bruker Solarix FTICR instrument equipped with a 7 T superconducting magnet. The transient duration was 0.4194 s, with an ion accumulation time of 0.025 s, and an average of 256 scans. These acquisition parameters were optimized for the low mass range (<300 Da) setting. We used the Bruker software (Bruker Compass Data Analysis 5.0 SRI x64) for Fourier transform and peak picking. The data was then exported to a computer for the application of our method. Other instrument settings are given in the Supporting Information Table 1.

## 5 Results and Discussion

Unless stated otherwise, we applied the standard isotope filtering parameters as given in the methods section.

### 5.1 Assignments in the Absence of Mass Error for a Perfectly Calibrated Data Set

We begin by analyzing the perfectly calibrated synthetic data set from Green and Perdue^17^. Figure 3 illustrates the mass deviation of all possible assignments, up to the limit of one ppm, as a function of *m*/*z*. Correct assignments are limited to the baseline (at zero ppm). A distinct, regular, almost symmetric line pattern is observed, asymptotically converging toward the baseline. Each line corresponds to a family of compounds sharing a specific LMM in the Diophantine solution leading to a fixed, exact mass deviation with respect to the baseline. Based on the special and the homogeneous Diophantine equations, LMMs can be added or subtracted, resulting in molecular formulas that appear symmetrically positioned with respect to the baseline. A small degree of asymmetry is due to constraints from the seven golden rules.

**Figure 3:**
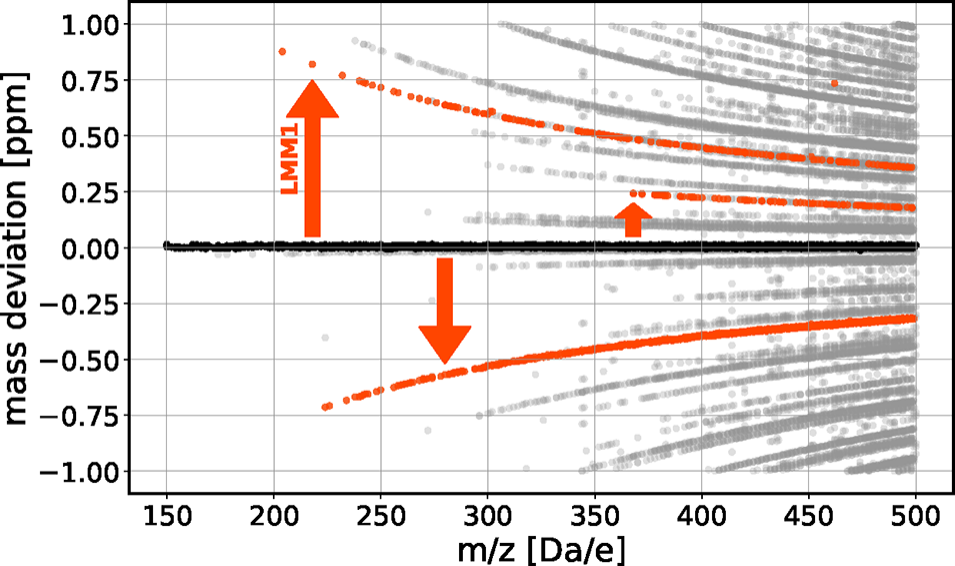
Mass deviation (within ±1 ppm) of all candidate molecular formulas for the perfectly calibrated synthetic data set of Green and Perdue^17^ as generated by the Diophantine solutions up to *m*/*z* = 500. We applied the elemental restrictions as given by the data (CHONPS). We applied the seven golden rules. Black dots denote correct assignments from the target list; other dots are incorrect candidate assignments. Each line represents a compound family obtained by adding or subtracting a specific LMM from a baseline formula, producing a fixed exact mass deviation. Three such families are highlighted in red, e.g. LMM1 = C_7_H_–2_O_–8_NS with ΔM = 1.76 × 10^–4^ Da.

### 5.2 Assignments in the Presence of Gaussian Mass Error

Figure 4 shows possible assignments generated by our algorithm for data with imposed Gaussian mass error with standard deviations of 0.05, and 0.09 ppm. In the graph, increasing the mass error reduces the resolution of the initial pattern, however, up to this level of mass inaccuracy, the pattern does not vanish.

**Figure 4:**
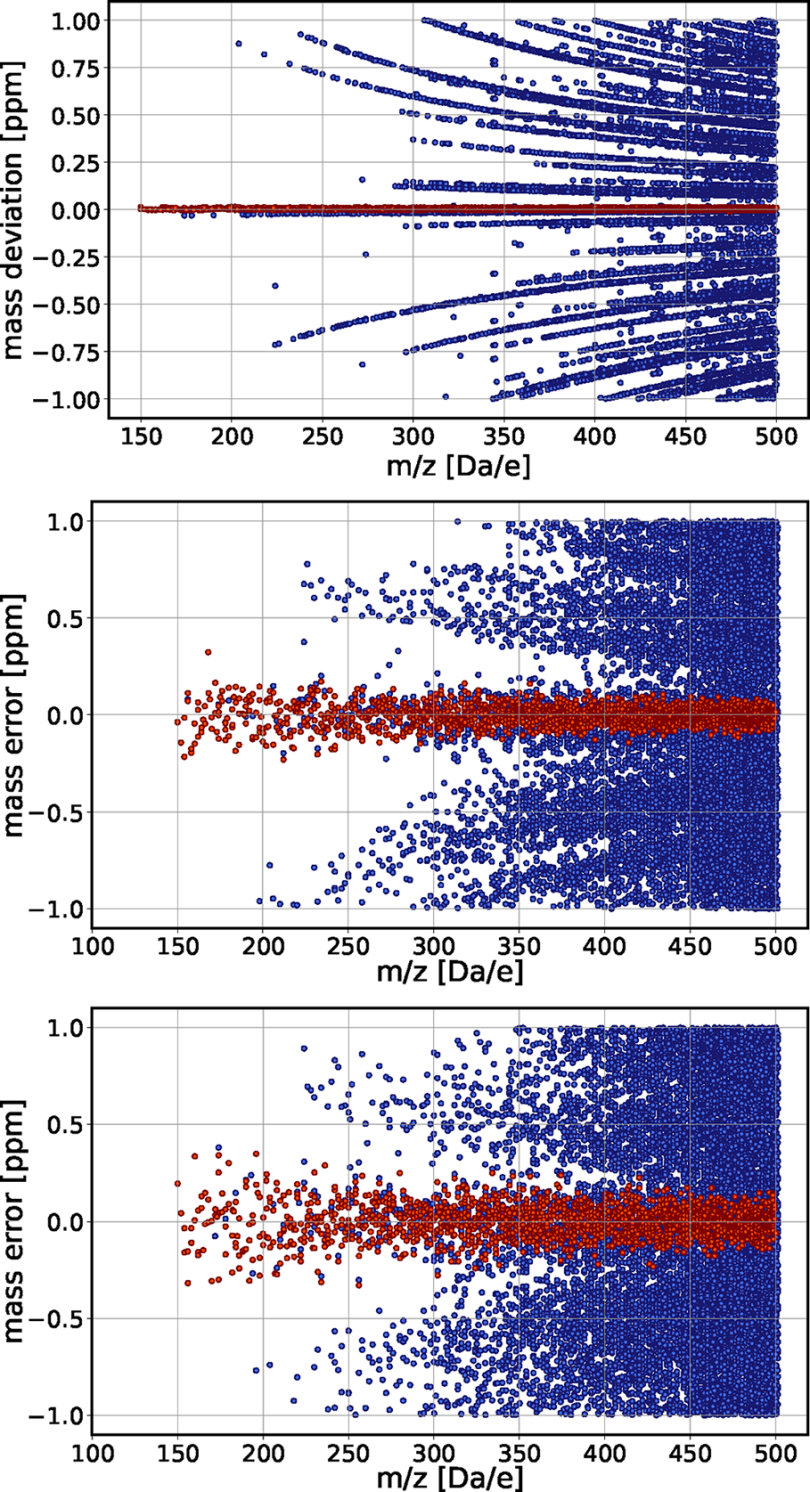
Mass deviation (within ±1 ppm) of all candidate molecular formulas for the perfectly calibrated synthetic data set of Green and Perdue^17^ as generated by the Diophantine algorithm up to *m*/*z* = 500. Top panel: perfectly calibrated data set without error (Figure 3). Middle and bottom panels: the same data set with imposed random Gaussian mass error of 0.05 and 0.09 ppm. Correct assignments from target mass list as performed by our algorithm are in red; alternative candidate formulas in blue. A higher level of mass error reduces the sharpness of the pattern but maintains the symmetry relative to the baseline.

We observe that the presence of mass error increases the total number of assignments (the number of blue points in Figure 4) from 13,280 in the ideal (noise free and perfectly calibrated data set to 13,630 at 0.09 ppm mass error. To assess the quality of the assignment, we determine the percentage of correctly identified formulas from the target mass list (150–500 Da) as a function of mass error (Figure 5). At 0.86 ppm mass error (a very high mass inaccuracy for UHRMS), approximately 66% of compounds, a substantial portion — 1172 out of 1789 — were still correctly identified.

**Figure 5:**
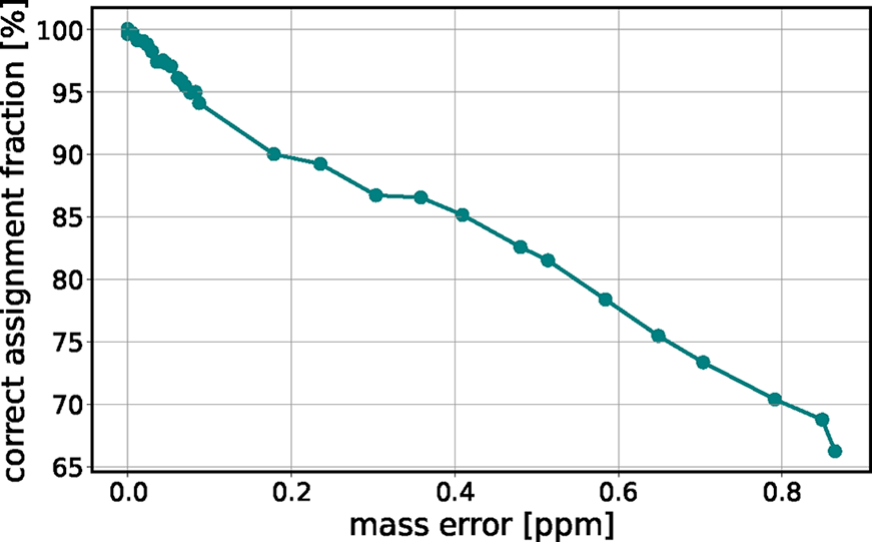
Percentage of correctly assigned compounds of the target list as a function of the random mass error imposed on the data. As the mass error increases, the ability to accurately identify compounds diminishes, resulting in fewer correct identifications, i.e., the number of red points in Figure 4 decreases as the mass error increases. Dots represent data from simulations; line is a guide for the eye.

Approximately 34% of the target mass list (608 out of 1789) consists of polyisotopic pairs. As long as mass error is not considered in isotope filtering, these compounds remain poorly identified (Figure 6). At 0.86 ppm mass error, 90% of polyisotopic compounds are lost. However, since missed polyisotopic pairs are reassigned to molecules with ^12^C, only half of these molecules are lost for correct assignment. This results in an effective 15% (half of 34% × 90%) decrease in correct assignment.

**Figure 6:**
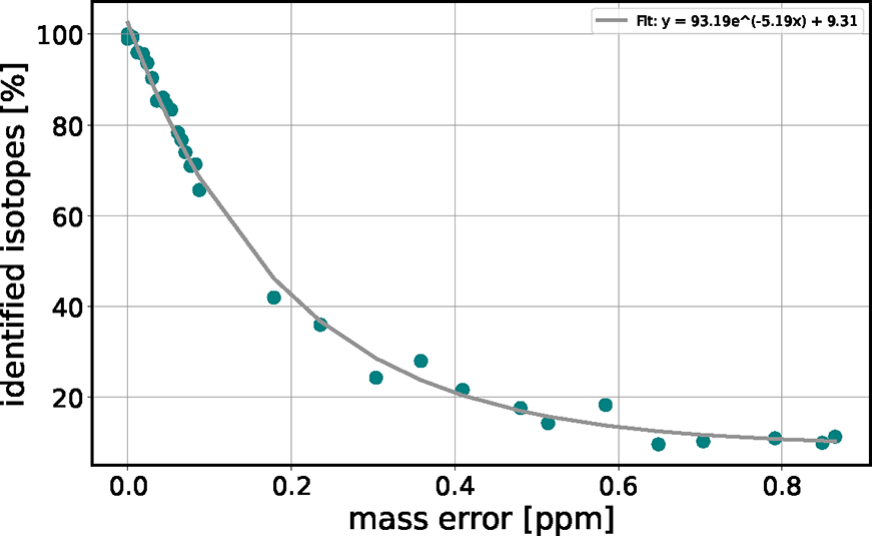
Percentage of correctly identified isotope pairs as a function of mass error, using the default parameters for isotope identification. As the mass error rises, the number of correctly identified isotopes decreases, subsequently affecting the assignments. Dots represent data points. The gray line represents an exponential function as fitted to the data.

The identification of isotopes for the synthetic data set is a two-step process, allowing for two potential improvements in the presence of mass error: (i) broadening the confidence limit for polyisotopic pairs by increasing Δ*M* (Figure 7) and (ii) relaxing the precision of the Kendrick mass defect (KMD) considering identical masses up to three decimal places instead of four.

**Figure 7:**
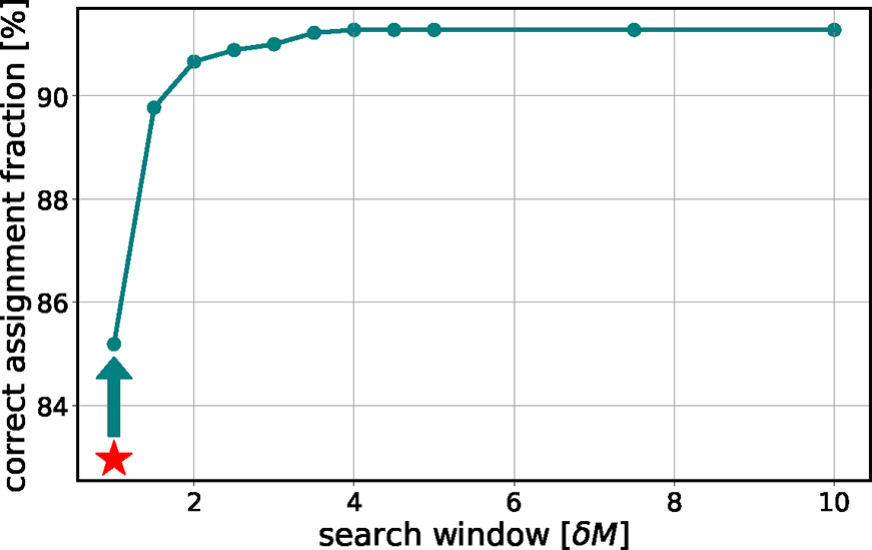
Percentage of correctly assigned isotopes of the target list at 0.5 ppm mass error as a function of multiples of the default isotope search window (δ*M* = 0.0001678 Da). The line is a guide for the eye. The star marks results using default parameters (four-decimal precision in KMD analysis); other points correspond to three-decimal precision in KMD. Beyond a search window four times the default, the gain is minor. We conclude that the isotope search window needs to be adjusted as a function of the mass error of the data.

In the following, as an example, we set the mass error of the data set to 0.5 ppm. We see (Figure 7) that the default parameters correctly identified 82% of the compounds (1458 out of 1789). Relaxing the KMD precision to three significant digits improved this to 1524, an increase of about 5%. Expanding the search interval by a factor of 4 while maintaining three-decimal KMD precision increased the correct identifications to 1633, resulting in a 12% improvement. Increasing the confidence limit beyond this threshold did not yield significant gains, but extended the computational time.

Based on the cumulative distribution function of a Gaussian, approximately 5% of assignments fall outside 2σ and remain unidentified. Since we limit ourselves to a deviation of 1 ppm, the theoretical maximum for correct assignments at a mass error of 0.5 ppm, is 95% (1699 compounds). With 1633 identified compounds (91.5%), we approach this limit rather well. Figure 8 illustrates the limit across different levels of mass error and the fraction of correct assignments (using the default parameter set). The relationship between the two is nontrivial with the fraction of correct assignments varying with mass error levels. The fraction of correct assignments remains bounded, however, by the cumulative Gaussian distribution if we limit ourselves to assignments within ±1 ppm.

**Figure 8:**
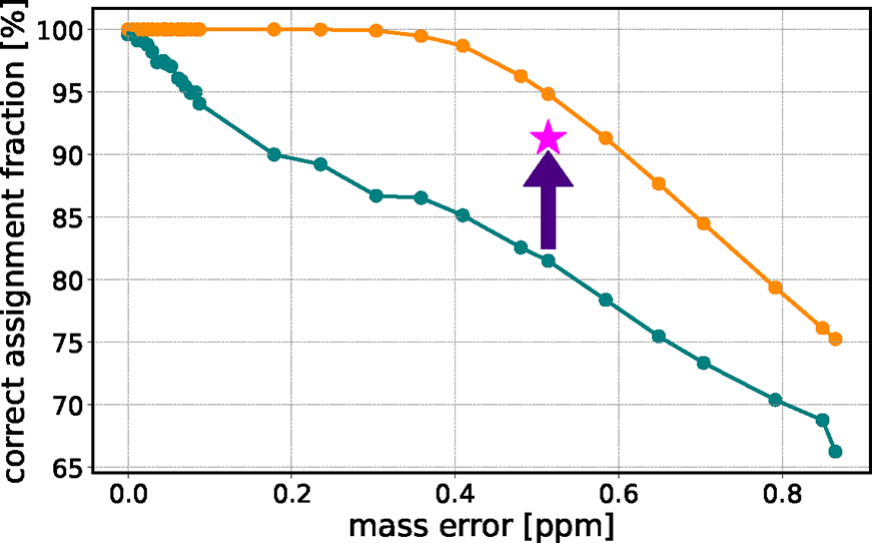
Percentage of correctly assigned components as a function of input mass error. The teal dots represent the identifications using the default parameter set. The orange dots represent the upper limit of accuracy according to the cumulative distribution function of a Gaussian. Refining the isotope filtering function increases the number of correctly assigned components by about 12% percent (arrow and star).

#### 5.2.1 Detecting Ill-Calibrated Data

We test our method on the synthetic data set with imposed random mass error that we decalibrated (see methods for details). The assignment process used the optimized parameters for isotope detection; i.e., a 4Δ*M* isotope search interval and the (relaxed) Kendrick Mass Defect (KMD) precision to the third significant digit. Results are shown in Figure 9. The decalibration function is directly visible from the shift of the entire pattern.

**Figure 9:**
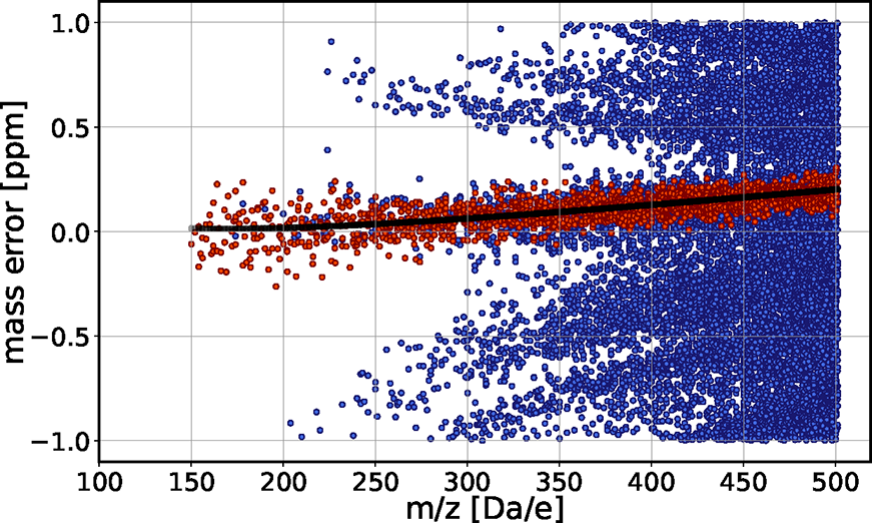
Mass deviation of all possible assignments within ±1 ppm from a decalibrated data set, subject to a random mass error of 0.05 ppm. Black dots represent the decalibrated data set without random mass error. Red points correspond to correctly identified masses from the target mass list. The pattern is asymmetrically distributed around the baseline, closely following the decalibration function.

### 5.3 Application to Experimental Data

We produced a prebiotic soup made from CHON (for details on production, see^6,49^ ). We added internal calibrants (listed in Table 3-SI). The instrument, a 7 T Bruker Solarix FTICR, was calibrated before measurement according to specifications from the manufacturer. We performed direct injection using ESI (see Methods for further details). Figure 10 (top) shows the mass deviations of possible CHON assignments from the measured mass values. The emerging pattern, however, does not align with the baseline, suggesting the presence of systematic error.

**Figure 10:**
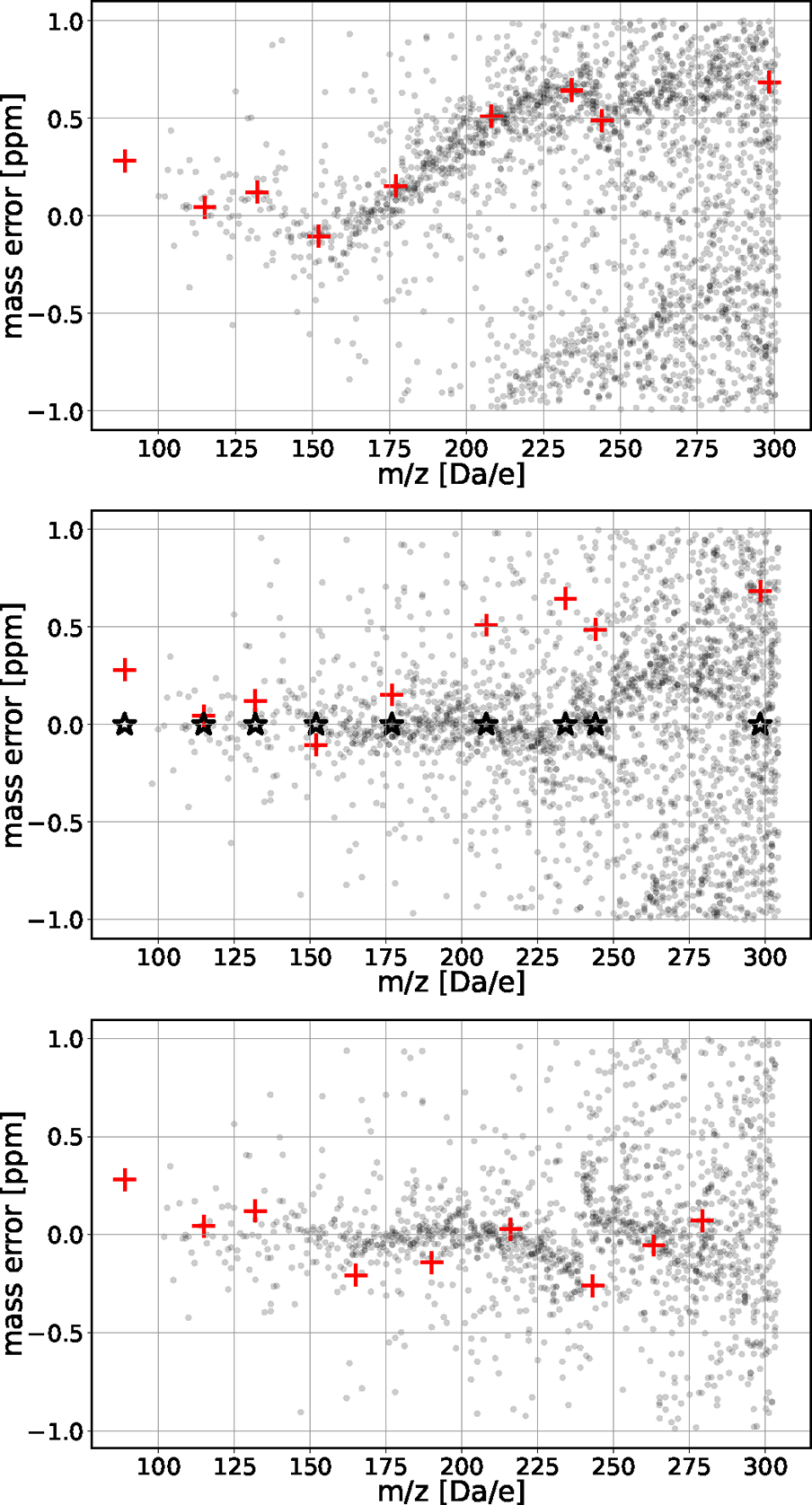
Mass deviation of all possible assignments within ± 1 ppm of deviation for a mass spectrum from a prebiotic molecular broth. The top panel represents the raw data of the measurement. The middle panel represents the pattern after “recalibration” based on the internal calibrants, highlighted as orange plus signs before, and black stars after recalibration. The bottom panel shows a pattern resulting from a second recalibration using identified molecules (orange plus signs) from the populated pattern at positions where no internal calibrants were present (see Figure 2a-SI for the corresponding pattern after a first recalibration). The mass deviation of the identified molecules after recalibration corresponds to zero ppm (not shown).

To address this, we identified the internal calibrants (see SI Table 3) by matching their known chemical formulas to assigned entries in the data set and extracting their mass deviations for recalibration. These calibrants are highlighted as orange plus signs in Figure 10 (top). They align along the most densely populated line of the plot. We then recalibrated the data by dividing the data into segments that contain three calibrants each and fitting a quadratic function accross the calibrants. This approach is inspired by the classical quadratic calibration equation of Ledford and Gross^48^ while the segment-wise implementation is analogous to the “walking calibration” strategy described by Savory^41^. We detailed the process in the SI, Sec. 0.5. As shown in Figure 10 (middle), recalibration with the internal calibrants aligns the pattern along the baseline.

Building on this, we performed an additional recalibration step using six assigned compounds. We combined them with the previously used internal calibrants (sets 2 in SI Sec. 0.5). This further aligned the most populated line to the baseline and improved the symmetry of the pattern as shown in Figure 10, bottom. We give detailed information on the calibration process in the Supporting Information.

We quantified the degree of randomness in the deviation of the assignments along the baseline using the autocorrelation function (ACF), defined as

$$\mathrm{ACF}(k)=\frac{\sum_{i=1}^{N-k}r_{i}r_{i+k}}{\sum_{i=1}^{N}r_{i}^{2}}$$

where *r*_*i*_ represents the mass deviation from the baseline of the *i* ^th^ assigned mass and *k* denotes the lag. Figure 11 shows the results. The lower degree of autocorrelation in the recalibrated data confirms a more random pattern, indicating that systematic deviations were reduced.

**Figure 11:**
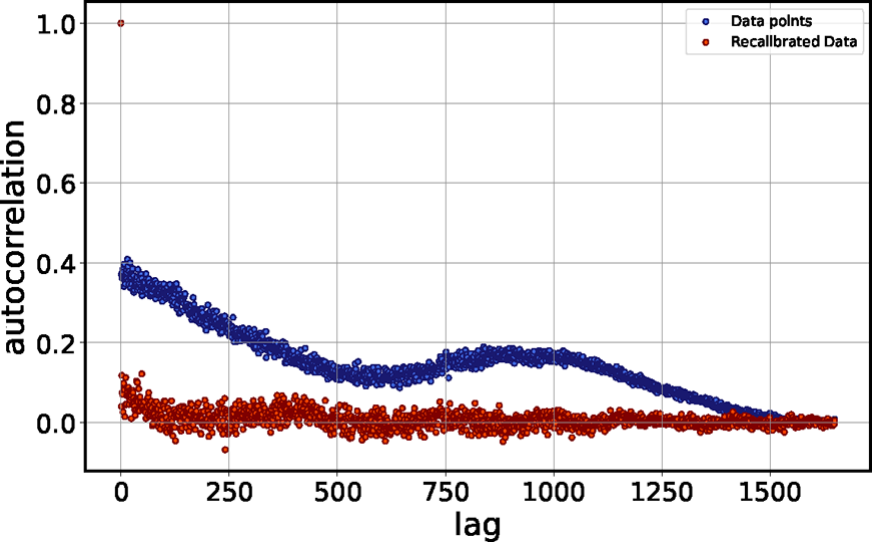
Autocorrelation function of mass deviations from the baseline: initial data (blue) and recalibrated data (orange). The reduced degree of autocorrelation in the recalibrated data confirms increased randomness of the appearing mass error. The lag corresponds to the number of successive data points taken as the “distance” that the autocorrelation coefficient is determined for.

In FT mass spectrometry, *m*/*z* values are determined by Fourier transform of the transient. If the transient is cut off early, this leads to a sinc shaped peak in the frequency domain. For transients that decay faster, however, the peak shape is Lorentzian (due to the exponential decay of the signal at the corresponding frequency). The peak shape corresponds to the uncertainty resulting from the limited data in the time domain. The *m*/*z* values are obtained by centroiding these peaks. Accordingly, the horizontal (*m*/*z*) uncertainties must follow the same Lorentzian (or sinc) distribution, other errors neglected. If, like in our case, the observation time of the transients is relatively long, the distribution of the *m*/*z* errors is expected to retain a Lorentzian profile – as opposed to a Gaussian. This is because the central limit theorem does not apply to Lorentzians. Note that others obtain similarly shaped distributions after recalibration^50^. However, it is clear that if the Lorentzian shape is lost, either due to very low amounts of ions or elevated amounts leading to a sinc function, the average resulting error should appear closer to a Gaussian.

To determine whether a Gaussian or Lorentzian function better fits the mass deviations after recalibration, we applied the Akaike Information Criterion (AIC) test (see SI Sec. 0.5 for details). Only after recalibration did the assignments clearly favor the Lorentzian distribution, consistent with visual inspection (Figure 12).

**Figure 12:**
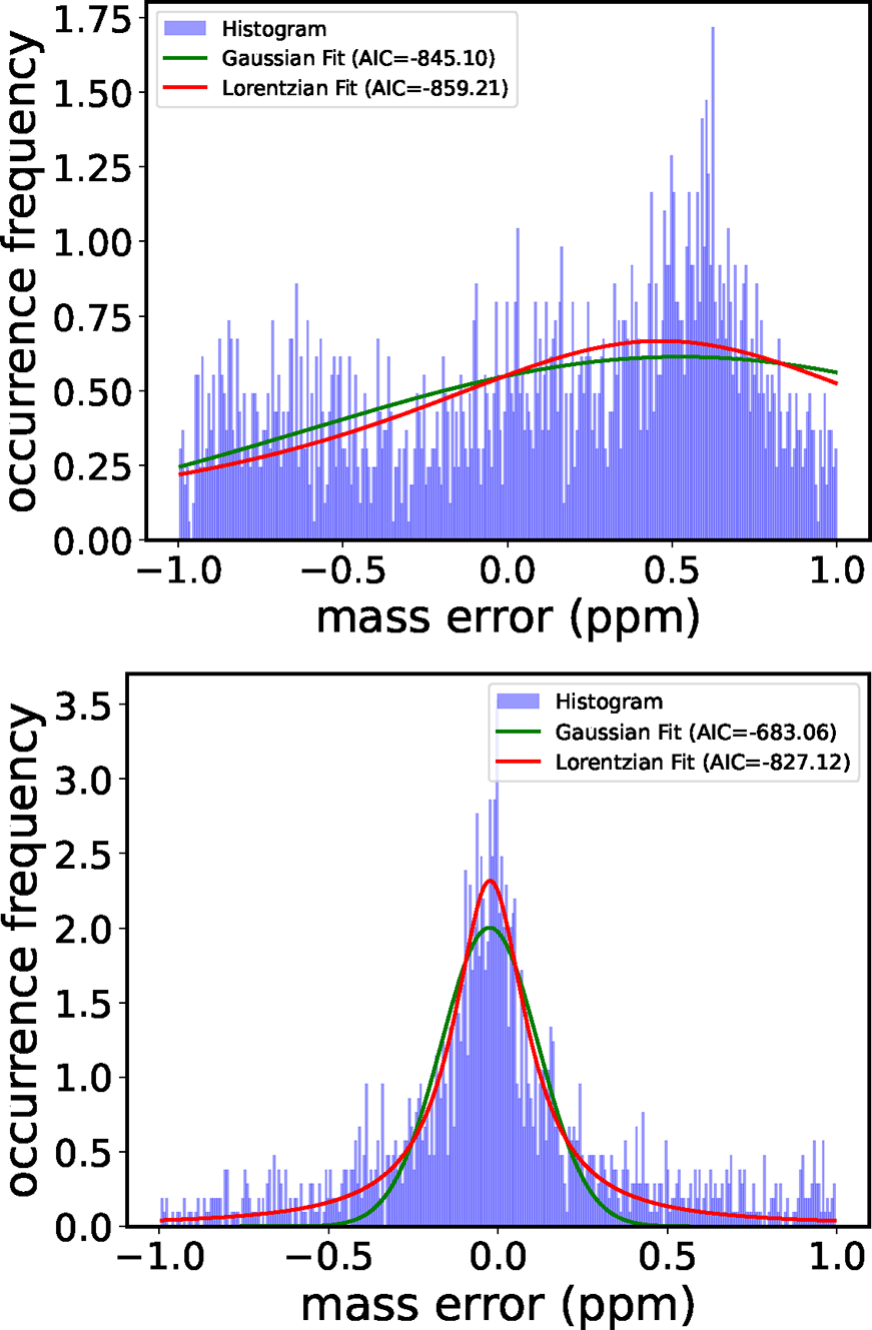
Histogram of mass deviations of all possible assignments based on raw and recalibrated data (see Figure 10, top and bottom panels). The histogram is constructed using a bin size of 0.5 Da (300 bins over the 150–300 Da range). Compared to a Gaussian distribution, the Lorentzian provides a better fit to the recalibrated data. This is confirmed by the AIC values (insert).

Note that the half width at half-maximum (HWHM) of the Lorentzian fit to the mass deviation distribution is beyond 0.2 ppm (See Figure 12). The calibration error appears negligible compared to this mass error. This becomes clear already in Figure 10, bottom, where the deviation of the most populous line from the baseline is smaller than the deviations cause by the mass error. In^50^, the authors achieved even slightly narrower error at about double *m*/*z*, however, this was at the expense of a much more sophisticated algorithm and data calibration correction and for a different sample. It will be interesting to see in future work, if our approach can be improved to reach similar statistics.

In our analysis of the synthetic data above, we focused on a Gaussian mass error distribution, which is not what we find in Figure 12. However, since the distribution function of the Lorentzian is limited by the noise floor, in practice, this function is finite. Accordingly we do not expect any principal differences in transferring our results to that case.

Given the results above, an automatic recalibration strategy could consist of identifying the most populous regions in the mass deviation plots by an edge detection algorithm (see SI for examples), and using the edges for recalibration. However, we reserve a detailed study about optimization and automation for future work. Many details, beyond the scope of the here presented work, remain to be investigated. Alternative strategies— such cross-correlation to establish the link between the theoretical and the experimentally observed pattern — may enhance assignment precision.

Moreover, one can feed our algorithm with improved data along already established directions. Our approach could be performed in Fourier space, where a gain in resolution can be achieved, in principle up to a factor of 2 ^51^. A further aspect to consider is the influence of concentration as reflected by peak intensity. Highly concentrated molecules generate much longer transients, leading to more intense and narrow Lorentzians. This means that, in principle, these should have a stronger weight in the calibration and attribution process. However, at the same time they tend to deviate more in *m*/*z* due to their increased space charge in the synchrotron. However, this dependence can be quantified and corrected for^26^. In the past, improved experimental approaches have been proposed. One of them is the OCULAR technique^32^, where scanning the entire mass range as small sections defined by a Quadrupole could greatly increase the precision of the *m*/*z* measurement while substantially increasing the number of detected substances as part of a highly complex mixture. The latter would improve our method, since more data is available for recalibration. At the same time it is a segment-wise measurement, which fits well to our segment-wise recalibration strategy. Moreover, better statistics on chemically meaningful molecular formulas that go beyond the seven golden rules could improve the assignments further.

## 6 Conclusions

Here we presented a novel method for assigning chemical formulas to mass spectrometry data in a combinatorial approach. Using linear Diophantine equations and corresponding low-mass moieties (LMMs), we looked at the ensemble of possible attributions of chemically plausible combinations within a given accuracy. Plotting mass deviations as a function of *m*/*z*, this generated a symmetric line pattern in a perfectly calibrated set. The symmetry stems from the low mass moieties that appear as general solutions to the corresponding homogeneous Diophantine equations. The symmetry was used for recalibrating the data. Recalibration yielded a Lorentzian mass error spectrum as expected from the physical limitations of the instrument. This spectrum quantifies the level of random mass error, an information that is crucial to achieve predictable likelihoods of correct assignments in a consistent approach. Note that at the same time our method delivers an excellent tool to optimize the settings of an instrument, since the mass inaccuracy becomes easily quantifiable.

## Supplementary Material



## Notes

Detailed solution to the homogeneous Diophantine equation, including the underlying vector space and calculation of a basis; summary of the experimental parameters as used in FTICR in a table; exact masses used in this study; description of the isotopologue filtering algorithm, supported by an exemplary calculation; description of the segmentwise recalibration process, supported by additional figures showing recalibration performance and a table of calibrants used; visualization of mass deviation patterns using edge detection; and supplementary references (PDF)

## References

[1] Cooper W. T., Chanton J. C., D’Andrilli J., Hodgkins S. B., Podgorski D. C., Stenson A. C., Tfaily M. M., Wilson R. M. (2022). A history of molecular
level analysis of natural organic matter by FTICR mass spectrometry
and the paradigm shift in organic geochemistry. Mass Spectrom. Rev..

[2] Qi Y., Fu P., Volmer D. A. (2022). Analysis of natural organic matter via fourier transform
ion cyclotron resonance mass spectrometry: an overview of recent non-petroleum
applications. Mass Spectrom. Rev..

[3] Schum S. K., Zhang B., Džepina K., Fialho P., Mazzoleni C., Mazzoleni L. R. (2018). Molecular
and physical characteristics of aerosol at
a remote free troposphere site: implications for atmospheric aging. Atmospheric Chemistry and Physics.

[4] Mazzoleni L. R., Ehrmann B. M., Shen X., Marshall A. G., Collett J. L. (2010). Water-soluble
atmospheric organic matter in fog: exact
masses and chemical formula identification by ultrahigh-resolution
Fourier transform ion cyclotron resonance mass spectrometry. Environ. Sci. Technol..

[5] Bianco A., Sordello F., Ehn M., Vione D., Passananti M. (2020). Degradation
of nanoplastics in the environment: Reactivity and impact on atmospheric
and surface waters. Sci. Total Environ..

[6] Wollrab E., Scherer S., Aubriet F., Carré V., Carlomagno T., Codutti L., Ott A. (2016). Chemical analysis
of
a “Miller-type” complex prebiotic broth: Part I: Chemical
diversity, oxygen and nitrogen based polymers. Origins of Life and Evolution of Biospheres.

[7] Collins B. C., Hunter C. L., Liu Y., Schilling B., Rosenberger G., Bader S. L., Chan D. W., Gibson B. W., Gingras A.-C., Held J. M. (2017). Multi-laboratory
assessment
of reproducibility, qualitative and quantitative performance of SWATH-mass
spectrometry. Nat. Commun..

[8] Poulos R. C., Hains P. G., Shah R., Lucas N., Xavier D., Manda S. S., Anees A., Koh J. M., Mahboob S., Wittman M. (2020). Strategies
to enable large-scale proteomics for reproducible
research. Nat. Commun..

[9] Ghosh T., Philtron D., Zhang W., Kechris K., Ghosh D. (2021). Reproducibility
of mass spectrometry based metabolomics data. BMC Bioinf..

[10] Hawkes J. A., d’Andrilli J., Agar J. N., Barrow M. P., Berg S. M., Catalán N., Chen H., Chu R. K., Cole R. B., Dittmar T. (2020). An international laboratory comparison of dissolved
organic matter composition by high resolution mass spectrometry: Are
we getting the same answer?. Limnology and Oceanography:
Methods.

[11] Zherebker A., Kim S., Schmitt-Kopplin P., Spencer R. G., Lechtenfeld O., Podgorski D. C., Hertkorn N., Harir M., Nurfajin N., Koch B. (2020). Interlaboratory comparison of humic substances compositional
space as measured by Fourier transform ion cyclotron resonance mass
spectrometry (IUPAC Technical Report). Pure
Appl. Chem..

[12] Corilo, Y. PetroOrg. software; Florida State University, 2014.

[13] Leefmann T., Frickenhaus S., Koch B. P. (2019). UltraMassExplorer: a browser-based
application for the evaluation of high-resolution mass spectrometric
data. Rapid Commun. Mass Spectrom..

[14] Fu Q.-L., Fujii M., Riedel T. (2020). Development
and comparison of formula
assignment algorithms for ultrahigh-resolution mass spectra of natural
organic matter. Anal. Chim. Acta.

[15] Tolic N., Liu Y., Liyu A., Shen Y., Tfaily M. M., Kujawinski E. B., Longnecker K., Kuo L.-J., Robinson E. W., Pasa-Tolic L. (2017). Formularity: software for automated formula assignment of natural
and other organic matter from ultrahigh-resolution mass spectra. Analytical chemistry.

[16] Sparkman, D. Informatics and mass spectral databases in the evaluation of environmental mass spectral data. In Comprehensive Environmental Mass spectrometry; Lebedev, A. T. , Ed.; ILM Publications, 2012; p 89.

[17] Green N. W., Perdue E. M. (2015). Fast graphically
inspired algorithm for assignment
of molecular formulae in ultrahigh resolution mass spectrometry. Analytical chemistry.

[18] Perdue E. M., Green N. W. (2015). Isobaric molecular formulae of C, H, and O: a view
from the negative quadrants of van Krevelen space. Analytical chemistry.

[19] Schum S. K., Brown L. E., Mazzoleni L. R. (2020). MFAssignR: Molecular formula assignment
software for ultrahigh resolution mass spectrometry analysis of environmental
complex mixtures. Environmental Research.

[20] Meija J. (2006). Mathematical
tools in analytical mass spectrometry. Anal.
Bioanal. Chem..

[21] Balabin R. M., Lomakina E. I. (2011). Support vector machine regression
(LS-SVM)—an
alternative to artificial neural networks (ANNs) for the analysis
of quantum chemistry data?. Phys. Chem. Chem.
Phys..

[22] Rojas-Cherto M., Peironcely J. E., Kasper P. T., van der Hooft J. J., de Vos R. C., Vreeken R., Hankemeier T., Reijmers T. (2012). Metabolite identification using automated
comparison
of high-resolution multistage mass spectral trees. Analytical chemistry.

[23] Marshall A. G., Hendrickson C. L., Jackson G. S. (1998). Fourier transform ion cyclotron resonance
mass spectrometry: a primer. Mass Spectrom.
Rev..

[24] Ledford E. B., Rempel D. L., Gross M. L. (1984). Space charge
effects in Fourier transform
mass spectrometry. II. Mass calibration. Anal.
Chem..

[25] Wong R. L., Amster I. J. (2007). Experimental evidence for space-charge effects between
ions of the same mass-to-charge in Fourier-transform ion cyclotron
resonance mass spectrometry. International journal
of mass spectrometry.

[26] Barry J. A., Robichaud G., Muddiman D. C. (2013). Mass recalibration
of FT-ICR mass
spectrometry imaging data using the average frequency shift of ambient
ions. J. Am. Soc. Mass Spectrom..

[27] Easterling M. L., Mize T. H., Amster I. J. (1999). Routine
part-per-million mass accuracy
for high-mass ions: space-charge effects in MALDI FT-ICR. Analytical chemistry.

[28] Kharchenko A., Vladimirov G., Heeren R. M., Nikolaev E. N. (2012). Performance of Orbitrap
mass analyzer at various space charge and non-ideal field conditions:
simulation approach. Journal of The American
Society for Mass Spectrometry.

[29] Gorshkov M. V., Fornelli L., Tsybin Y. O. (2012). Observation of ion
coalescence in
Orbitrap Fourier transform mass spectrometry. Rapid Commun. Mass Spectrom..

[30] Aizikov K., Mathur R., O’connor P. B. (2009). The spontaneous loss of coherence
catastrophe in Fourier transform ion cyclotron resonance mass spectrometry. J. Am. Soc. Mass Spectrom..

[31] Nakata M. T., Hart G. W., Peterson B. G. (2010). Peak coalescence,
spontaneous loss
of coherence, and quantification of the relative abundances of two
species in the plasma regime: particle-in-cell modeling of Fourier
transform ion cyclotron resonance mass spectrometry. J. Am. Soc. Mass Spectrom..

[32] Lozano D. C. P., Gavard R., Arenas-Diaz J. P., Thomas M. J., Stranz D. D., Mejía-Ospino E., Guzman A., Spencer S. E., Rossell D., Barrow M. P. (2019). Pushing
the analytical limits: new insights into complex
mixtures using mass spectra segments of constant ultrahigh resolving
power. Chem. Sci..

[33] Kaur P., O’Connor P. B. (2004). Use of statistical methods for estimation of total
number of charges in a mass spectrometry experiment. Anal. Chem..

[34] Aizikov K., O’Connor P. B. (2006). Use of the filter diagonalization method in the study
of space charge related frequency modulation in Fourier transform
ion cyclotron resonance mass spectrometry. J.
Am. Soc. Mass Spectrom..

[35] Mathur R., Knepper R. W., O’Connor P. B. (2007). A low-noise,
wideband preamplifier
for a Fourier-transform ion cyclotron resonance mass spectrometer. J. Am. Soc. Mass Spectrom..

[36] Du P., Stolovitzky G., Horvatovich P., Bischoff R., Lim J., Suits F. (2008). A noise model
for mass spectrometry based proteomics. Bioinformatics.

[37] Kwon D., Vannucci M., Song J. J., Jeong J., Pfeiffer R. M. (2008). A novel
wavelet-based thresholding method for the pre-processing of mass spectrometry
data that accounts for heterogeneous noise. Proteomics.

[38] Urban, J. ; Štys, D. Noise and baseline filtration in mass spectrometry. In Bioinformatics and Biomedical Engineering: Third International Conference, IWBBIO 2015; Granada, Spain, 2015; pp 418–425.

[39] Shin H., Mutlu M., Koomen J. M., Markey M. K. (2007). Parametric power
spectral density analysis of noise from instrumentation in MALDI TOF
mass spectrometry. Cancer Inform..

[40] Frevel L. K., Lee W.-L., Tecklenburg R. E. (1999). Diophantine
mass spectrometric structure
analysis. J. Am. Soc. Mass Spectrom..

[41] Savory J. J., Kaiser N. K., McKenna A. M., Xian F., Blakney G. T., Rodgers R. P., Hendrickson C. L., Marshall A. G. (2011). Parts-per-billion
Fourier transform ion cyclotron resonance mass measurement accuracy
with a “walking” calibration equation. Analytical chemistry.

[42] Makarov A., Denisov E., Lange O., Horning S. (2006). Dynamic range of mass
accuracy in LTQ Orbitrap hybrid mass spectrometer. J. Am. Soc. Mass Spectrom..

[43] Qi Y., Barrow M. P., Van Orden S. L., Thompson C. J., Li H., Perez-Hurtado P., O’Connor P. B. (2011). Variation of the Fourier transform
mass spectra phase function with experimental parameters. Analytical chemistry.

[44] Kind T., Fiehn O. (2007). Seven Golden Rules for heuristic filtering of molecular formulas
obtained by accurate mass spectrometry. BMC
Bioinf..

[45] Łacki M. K., Valkenborg D., Startek M. P. (2020). IsoSpec2: ultrafast
fine structure
calculator. Analytical chemistry.

[46] Łacki M. K., Startek M., Valkenborg D., Gambin A. (2017). IsoSpec: Hyperfast
fine structure calculator. Analytical chemistry.

[47] Gross, J. H. Massenspektrometrie: Ein Lehrbuch; Springer-Verlag, 2012.

[48] Ledford E., Ghaderi S., White R., Spencer R., Kulkarni P., Wilkins C., Gross M. (1980). Exact mass
measurement by Fourier
transform mass spectrometry. Anal. Chem..

[49] Ravanbodshirazi S., Boutfol T., Safaridehkohneh N., Finkler M., Mohammadi-Kambs M., Ott A. (2023). The nature of the spark
is a pivotal element in the design of a miller–urey
experiment. Life.

[50] Smith D. F., Kharchenko A., Konijnenburg M., Klinkert I., Paša-Tolić L., Heeren R. M. (2012). Advanced mass calibration and visualization for FT-ICR
mass spectrometry imaging. Journal of the American
society for mass spectrometry.

[51] Sanders J. D., Butalewicz J. P., Clowers B. H., Brodbelt J. S. (2021). Absorption
Mode
Fourier Transform Ion Mobility Mass Spectrometry Multiplexing Combined
with Half-Window Apodization Windows Improves Resolution and Shortens
Acquisition Times. Analytical chemistry.

