# Supplementary material for: UHRMS Formula Assignment: Diophantine-Based Recalibration Yields Lorentzian Mass Error Distribution as the Limiting Factor
Source: J Am Soc Mass Spectrom. 2025 Dec 26;37(1):105–15. doi: 10.1021/jasms.5c00226 (PMC12784403; doi:10.1021/jasms.5c00226)
Supplement: Supplementary file 1 [file js5c00226_si_002.pdf]

Supplementary Information for:

UHRMS Formula Assignment: Diophantine-Based Recalibration Yields Lorentzian Mass Error Distribution as the Limiting Factor

Neda Safaridehkohneh and Albrecht Ott*

Saarland University, Dept of Physics, Center for Biophysics, 66123 Saarbrücken, Germany

E-mail: albrecht.ott@physik.uni-saarland.de

This document contains supplementary figures, tables, and methods supporting the main manuscript.

The Solutions to the Homogeneous Diophantine Equation Form a Vector Space, Calculation of a Base

We begin by considering the atomic species C, H, and O. The generalization to additional elements is addressed further down.

Let $C = 12$, $H = 1$, and $O = 16$ denote the nominal masses of C, H, and O, respectively. The condition for retaining nominal mass is given by the homogeneous Diophantine equation:

$$12c + 16o + h = 0 \tag{1}$$

Equation (1) defines a plane in $\mathbb{Z}^3$ normal to the vector $(12, 16, 1)$. We are interested in all integer solutions (c, o, h) lying on this plane. This is an infinite set of solutions (as long as there are no limits on c,o, and h).

A basis for this lattice is given by $\vec{x}$ and $\vec{y}$:

$$\vec{r} = s\vec{x} + t\vec{y}, \quad \text{where } \vec{x} = \begin{pmatrix} 1 \\ -12 \\ 180 \end{pmatrix}, \quad \vec{y} = \begin{pmatrix} 0 \\ 1 \\ -16 \end{pmatrix}, \quad s, t \in \mathbb{Z} \tag{2}$$

Generalization to Additional Elements

The above can easily be generalized to more atomic species. Adding nitrogen introduces a fourth component, n, with nominal mass $N = 14$. The mass balance condition becomes:

$$12c + 16o + 14n + h = 0 \tag{3}$$

A lattice basis of integer solutions in $\mathbb{Z}^4$ is:

$$\begin{pmatrix} c \\ o \\ n \\ h \end{pmatrix} = \begin{pmatrix} 1 & 0 & 0 \\ -12 & 1 & 0 \\ 180 & -16 & 1 \\ -2340 & 208 & -14 \end{pmatrix} \begin{pmatrix} t \\ s \\ r \end{pmatrix}, \quad r, s, t \in \mathbb{Z}$$

Any other valid basis spans the same lattice and can be obtained via a unimodular transformation. Let $\vec{x}, \vec{y}, \vec{z}$ be a known basis, and $\vec{a}, \vec{b}, \vec{c}$ a new basis. Then:

$$\begin{aligned} \vec{x} &= \lambda_x \vec{a} + \mu_x \vec{b} + \nu_x \vec{c} \\ \vec{y} &= \lambda_y \vec{a} + \mu_y \vec{b} + \nu_y \vec{c} \\ \vec{z} &= \lambda_z \vec{a} + \mu_z \vec{b} + \nu_z \vec{c} \end{aligned} \tag{4}$$

This defines a matrix M of integer coefficients. If $\det(M) = \pm 1$, the inverse also consists

of integers, and both bases are equivalent under a unimodular transformation[1].

Example Unimodular Base Transformation

A canonical unimodular matrix has the form:

$$\begin{pmatrix} 1 & n & m \\ 0 & 1 & l \\ 0 & 0 & 1 \end{pmatrix}, \quad n, m, l \in \mathbb{Z}$$

An example transformation using this matrix ($n = 2, m = 5, l = 4$) is:

$$\begin{pmatrix} \vec{a} \\ \vec{b} \\ \vec{c} \end{pmatrix} = \begin{pmatrix} \vec{x} + 2\vec{y} + 5\vec{z} \\ \vec{y} + 4\vec{z} \\ \vec{z} \end{pmatrix}$$

Such transformations can improve computational efficiency or align with chemically meaningful axes.

Solving Diophantine Equations

Two Variables

To solve:

$$a_1 x_1 + a_2 x_2 = 0$$

Assuming $\gcd(a_1, a_2) = 1$, the general solution is:

$$x_1 = t a_2, \quad x_2 = -t a_1, \quad t \in \mathbb{Z}$$

Example:

$$12c + h = 0 \Rightarrow c = t, \quad h = -12t$$

Three Variables

To solve:

$$a_1x_1 + a_2x_2 + a_3x_3 = 0$$

Let $b = \gcd(a_2, a_3)$. Then rewrite:

$$a_1x_1 + by = 0, \quad \text{where } a_2x_2 + a_3x_3 = by$$

Solve the 2-variable equations step-by-step:

$$x_1 = tb', \quad y = -ta_1'$$

Then solve $a_2x_2 + a_3x_3 = by$ using standard methods. The full solution is a linear combination of a particular and homogeneous solution.

Example:

$$12c + 16o + h = 0 \Rightarrow (c, o, h) = t(1, -12, 180) + s(0, 1, -16)$$

Four Variables

Apply a similar recursive strategy.

Given:

$$a_1x_1 + a_2x_2 + a_3x_3 + a_4x_4 = 0$$

Group and reduce the terms using GCDs, then build the basis via successive substitutions.

Final result for (c, o, n, h) is:

$$\begin{pmatrix} c \\ o \\ n \\ h \end{pmatrix} = t \begin{pmatrix} 1 \\ -12 \\ 180 = (12 \times (16-1)) \\ -2340 = -(12 \times (16-1) \times (14-1)) \end{pmatrix} + s \begin{pmatrix} 0 \\ 1 \\ -16 \\ 208 = (16 \times (14-1)) \end{pmatrix} + r \begin{pmatrix} 0 \\ 0 \\ 1 \\ -14 \end{pmatrix}$$

Higher Dimensions

To add a fifth element (e.g., sulfur), define a new vector:

$$\begin{pmatrix} \vdots \\ x \\ h \end{pmatrix} = q \begin{pmatrix} \vdots \\ 1 \\ -m_x \end{pmatrix}$$

The construction generalizes inductively.

Experimental Setup

Table 1: FTMS Instrument Adjusted Parameters

Category	Parameter	Value/Setting
General Settings	Size	1M
	Low m/z	100.35
	High m/z	300.00
	Avg Scans	40
	Accumulation Time (s)	0.25
	Polarity	Positive
	API High Voltage	Enabled
	Source Quench	Enabled
	Serial Mode	Disabled
API Source	Capillary Voltage	4500 V
	End Plate Offset	-500 V
Source Gas Tune	Nebulizer Pressure	2.5 bar
	Dry Gas Flow	6.0 L/min
	Dry Temperature	240°C
Ion Transfer – Source Optics	Capillary Exit	220.0 V
	Deflector Plate	200.0 V
	Funnel 1 Voltage	150.0 V
	Skimmer 1 Voltage	15.0 V
	Funnel RF Amplitude	150.0 Vpp
Ion Transfer – Octopole & Quadrupole	Frequency	5 MHz
	RF Amplitude	350.0 Vpp
	Q1 Mass	50.00 m/z
Collision Cell & Transfer Optics	Collision Voltage	-1.5 V
	DC Extract Bias	0.5 V
	RF Frequency	2 MHz
	Collision RF Amplitude	1600.0 Vpp
	Time of Flight	0.700 ms
Gas Control & Analyzer	Flow	32%
	Gas Control	Enabled
	Transfer Exit Lens	-14.0 V
	Analyzer Entrance	-7.0 V

Exact Masses

Table 2 presents the exact masses used in this calculation.

Table 2: List of Elements and their Exact Masses

Element	Symbol	Exact Mass (Da)
Carbon	C	12.000000
Carbon-13	^{13}C	13.003355
Hydrogen	H	1.007825
Oxygen	O	15.994915
Nitrogen	N	14.003074
Phosphorus	P	30.973763
Sulfur	S	31.972072

Isotope Filtering and Relative Intensity Analysis

Relative Intensity Analysis

Isotope filtering includes checking the ratio of the detected intensities that must match the expected ratio of paired isotopes.

Approximately 1.1% of naturally occurring carbon is carbon-13 (^{13}C), while the remaining 98.9% is carbon-12 (^{12}C). Consequently, in a mass spectrum, we expect to observe both a monoisotopic peak (containing ^{12}C atoms) and an isotopic peak (containing ^{13}C atoms) for any molecule that contains carbon.

For a molecule with C carbon atoms, a nominal mass M, the intensity of its monoisotopic peak I_M, there should be an isotopic peak with nominal mass $M + 1$ and an intensity I_{M+1}. The ratio of these intensities can be expressed as:

$$\frac{I_M}{I_{M+1}} = \frac{100}{1.1C}.$$

Taking natural logarithms of both sides, we obtain:

$$\ln\left(\frac{I_M}{I_{M+1}}\right) = \ln\left(\frac{100}{1.1C}\right).$$

This can be further simplified to:

$$\ln I_M - \ln I_{M+1} = \ln\left(\frac{100}{1.1C}\right).$$

We consider a ten-percentage error tolerance for the logarithm of the ratio of the detected intensities.

Isotope filtering: Example

Step 1. Search Interval

Define a search interval centered around the expected isotopic shift of $1.003355Da$, with a tolerance of $\pm 0.0001678Da$ as follows:

$$M_1' = M_1 + 1.003355 - 0.0001678 = 236.1838272$$
$$M_2' = M_1 + 1.003355 + 0.0001678 = 236.1841628$$

Upon searching the input mass list, only one mass was found to lie within this interval, i.e., $M_2 = 236.18399\ Da$.

Step 2. Kendrick Mass Defect Calculation

The Kendrick Mass Defect (KMD) is obtained as follows: For the initial mass:

$$(KMD)_M = \frac{M}{1.003355} - \left[\frac{M}{1.003355}\right]$$
$$(KMD)_{M_1} = \frac{235.18064}{1.003355} - \left[\frac{235.18064}{1.003355}\right]$$
$$= 234.394247 - 234 = 0.394247 \quad \text{(six decimal places)}$$

Similarly, for $M_2 = 236.18399\ Da$:

$$M_2 = 236.18399 \quad \Longrightarrow \quad (KMD)_{M_2} = 0.394242$$

Assigned Formula: $C_{14}H_{22}N_2O$ and $^{13}CC_{13}H_{22}N_2O$

Step 3. Relative Intensity Analysis The following intensities were detected:

$$I_1 = 4324507136 \quad I_2 = 677732800$$

The natural logarithmic difference is given by:

$$\ln I_1 - \ln I_2 = 1.8533$$

For comparison, the expected value is calculated as:

$$\ln \frac{100}{14 \times 1.1} = 1.870$$

The deviation is calculated as:

$$Deviation = \frac{1.870 - 1.8533}{1.870} \times 100 = 8.93\%$$

A deviation within ten percentage of the expected natural logarithm of the intensity ratios indicates a match. This threshold is adjustable based on instrument precision and experimental conditions.

Segmentwise Mass Recalibration Using Quadratic Functions

Three known compounds are selected to define a segment. These known compounds can be either internal calibrants injected into the sample prior to measurement or correctly assigned compounds from the primary assignment, chosen based on their relevance to the segment's characteristics.

The recalibration follows the quadratic equation:

$$ax_i^2 + bx_i + c = x_i'$$

where x_i and x_i' represent the measured and exact masses of the known compounds, respectively. Solving this equation for three known compounds determines the coefficients a, b, and c:

$$ax_1^2 + bx_1 + c = x_1'$$
$$ax_2^2 + bx_2 + c = x_2'$$
$$ax_3^2 + bx_3 + c = x_3'.$$

All measured masses within the given segment are then recalibrated using these coefficients. The assignment process is again applied after recalibration.

The distribution of mass deviations ($\Delta M = M_{\textbf{Measured Mass}} - M_{\textbf{Assigned Mass}}$) is fitted by Gaussian and Lorentzian function. The Akaike Information Criterion (AIC)[2], provides an additional measure to select the best recalibration.

Table 3 summarizes the calibrants used, including both internal standards and assigned compounds. The results of selected calibrant sets are presented below, followed by a summary of the AIC values in Table 4.

Table 3: Compounds Used as Calibrants

**Internal Calibrants**		
**Chemical Formula**	**Measured Mass**	**Exact Mass**
$C_{14}H_{22}ON_2$	234.173364	234.173213
$C_5H_9O_2N$	115.063334	115.063329
$C_{10}H_{16}O_3N_2S$	244.088284	244.088165
$C_5H_{12}O_2N_2$	132.089894	132.089878
$C_8H_7O_2N_3$	177.053854	177.053827
$C_{19}H_{38}O_2$	298.287384	298.28718
$C_3H_7O_2N$	89.047704	89.047679
**Assigned Compounds**		
$C_{11}H_{19}N$	165.151715	165.151749
$C_{12}H_{14}O_2$	190.099353	190.099380
$C_{12}H_{16}N_4$	216.137022	216.137496
$C_{15}H_{21}N_3$	243.173483	243.173546
$C_{15}H_{25}ON_3$	263.199747	263.199761
$C_{18}H_{21}N_3$	279.173567	279.173547
$C_9H_{12}O_2$	152.083714	152.08373
$C_{14}H_{12}N_2$	208.100154	208.100048
$C_{15}H_{18}$	198.140944	198.140850
$C_{15}H_{27}O_2N$	253.204344	253.204179
$C_{20}H_{28}N_2$	296.225474	296.225247

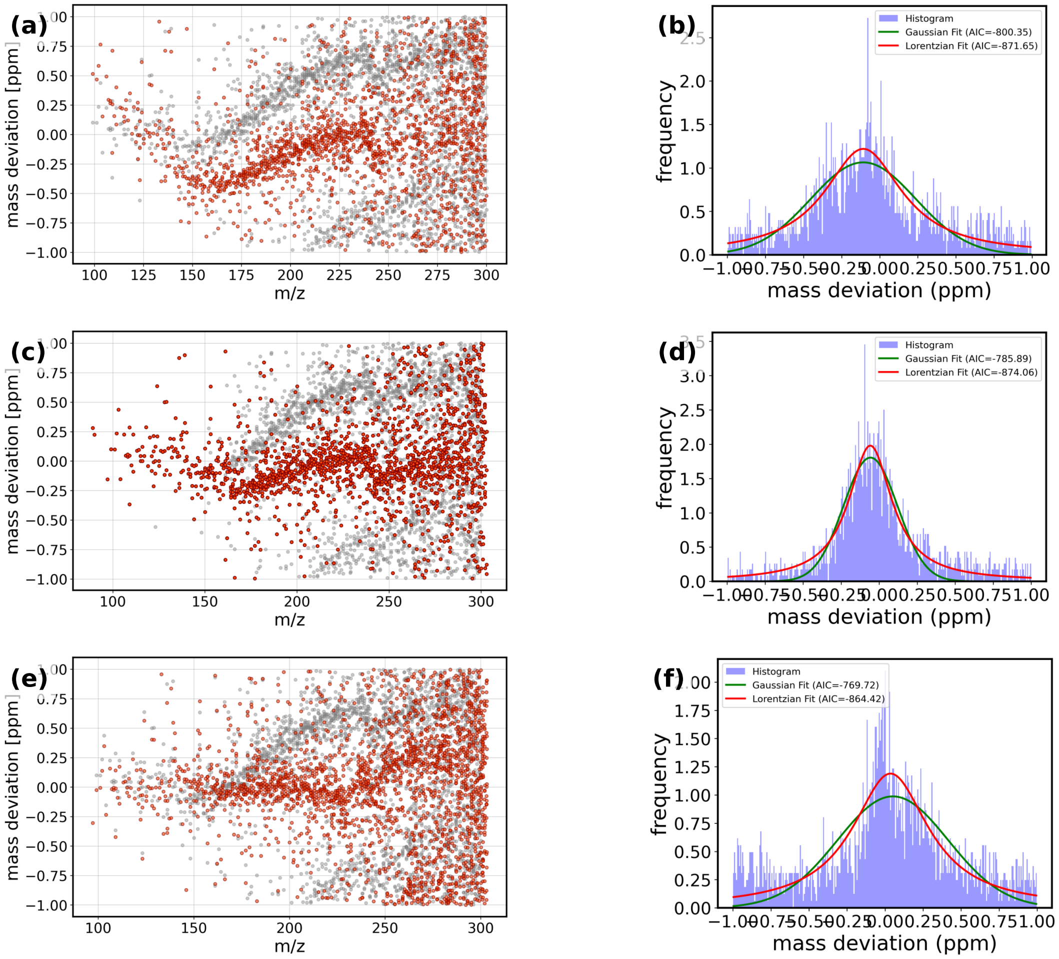

Figure 1: Mass deviation (left) before (gray) and after (orange) recalibration and distribution of mass deviation (right) after one-step recalibration for three calibrant sets. Panels a,b (set 1): calibrants at nominal masses 132, 234, 298; c,d (set 2): 198, 253, 296; e,f (set 3): 89, 115, 132, 152, 177, 209, 234, 244, 298. Exact calibrant masses are listed in Table 3. Results from set 2 (panels c,d) were used as the starting point for a second recalibration shown in Figure 2.

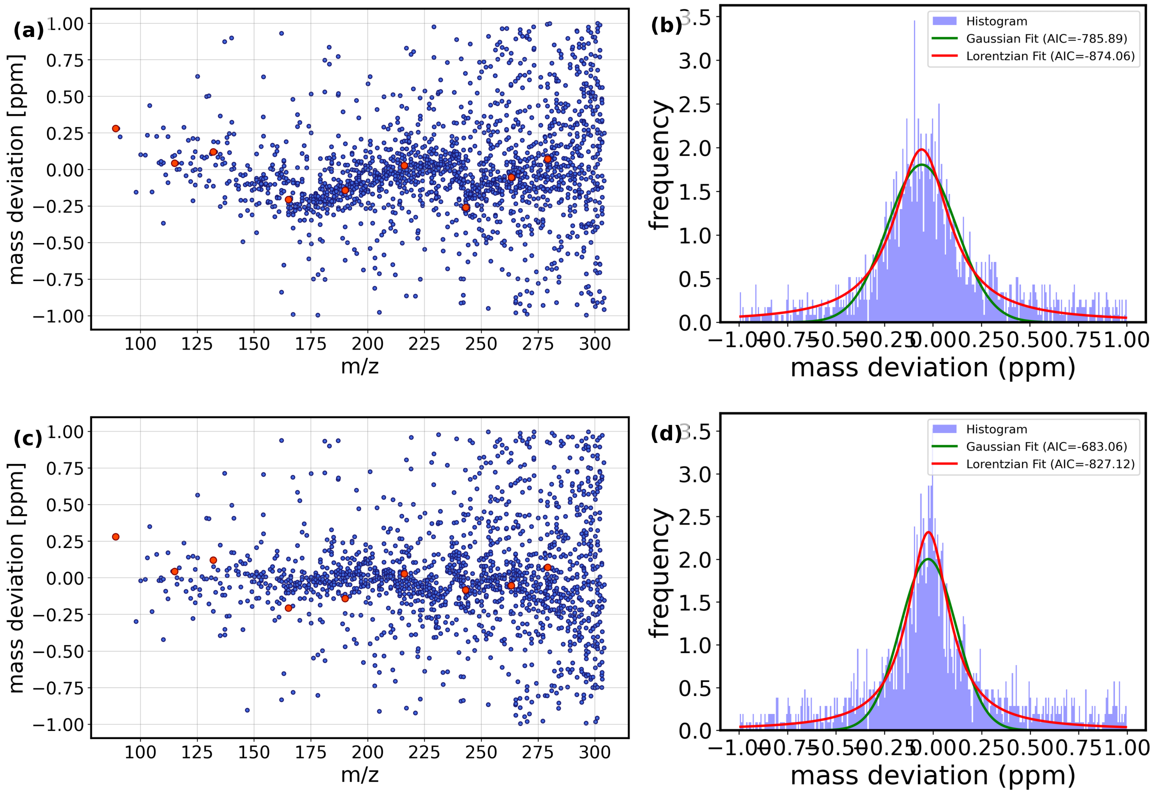

Figure 2: Mass deviation (left) and distribution of mass deviation (right) after iterative recalibration. Panels a,b (from Figure 1) show one-step recalibration using set 2 shown as reference; red dots mark calibrant positions. Panels c,d show results after a second recalibration using internal calibrants (89, 115, 132) and assigned compounds (165, 190, 216, 243, 263, 279), set 4. Lorentzian (red) and Gaussian (green) fits are applied to the distributions.

Table 4: Akaike Information Criterion (AIC) comparison of Lorentzian vs. Gaussian fits to full-range mass deviation data (100–300 Da) across four calibrant sets. Lower AIC values indicate consistently better performance of the Lorentzian model.

Calibration Set	Deviation Range	Gaussian AIC	Lorentzian AIC	Best Fit
Set 1	±1 ppm	-800.35	-871.65	Lorentzian
Set 2	±1 ppm	-785.89	-874.06	Lorentzian
Set 3	±1 ppm	-769.72	-864.42	Lorentzian
Set 4	±1 ppm	-683.06	-827.12	Lorentzian

Visualization of Mass Deviation Patterns Using Heatmaps

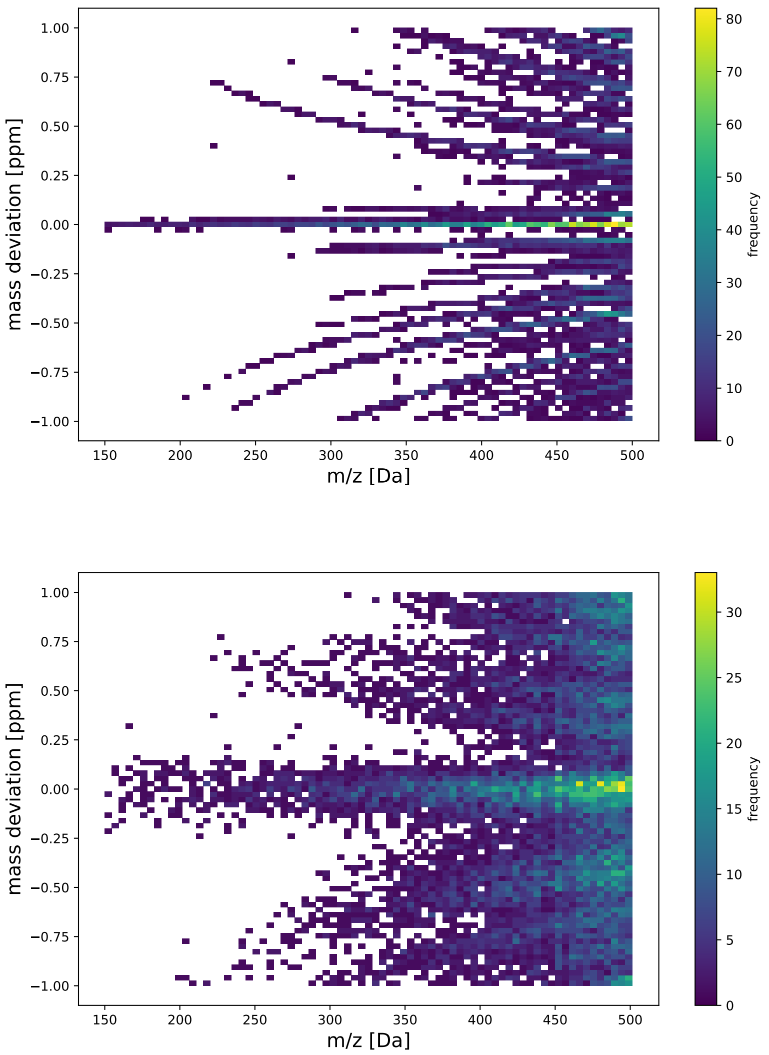

Figure 3: Heatmaps of mass deviation versus m/z generated using the Seaborn package. Deviations were normalized to [0, 1] and mapped to grayscale images along the m/z axis. Color intensity in the 2D histograms reflects the frequency of points per bin: bright regions indicate systematic patterns; sparse areas reflect noise or infrequent matches. **Top:** Synthetic dataset showing distinct, convergent line patterns, with the brightest line denoting the baseline. **Bottom:** Same dataset with 0.05 ppm Gaussian noise; line features broaden into bands, but the underlying pattern remains visible in high-intensity regions.

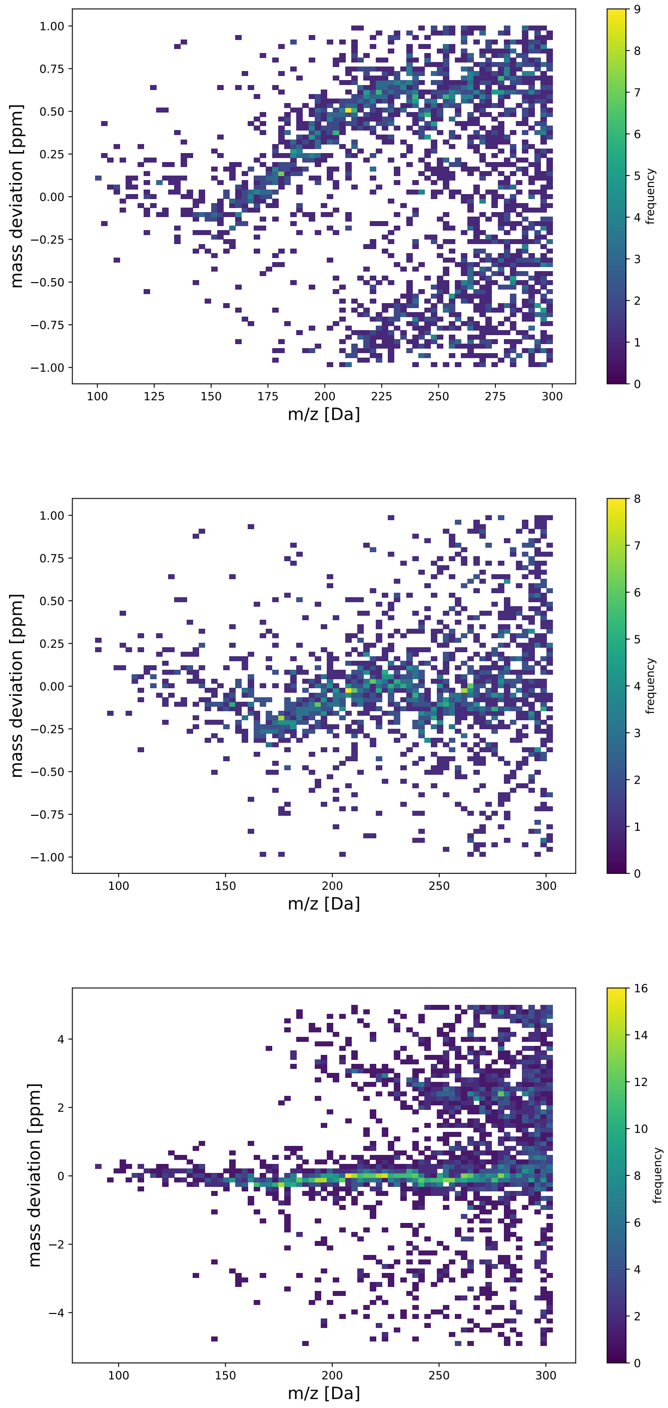

Figure 4: Heatmaps of mass deviation versus m/z for the experimental dataset. Deviations were normalized to [0, 1] and visualized as 2D histograms using the Seaborn package, where color intensity reflects point density. **Top:** Data before recalibration, showing widespread deviation. **Middle and bottom:** After recalibration at two visualization scales; denser, well-aligned features indicate improved assignment.

References

(1) Hanson, R. Integer matrices whose inverses contain only integers. *The Two-Year College Mathematics Journal* **1982**, *13*, 18–21.

(2) Akaike, H. A new look at the statistical model identification. *IEEE transactions on automatic control* **1974**, *19*, 716–723.
